# Supplementary material for: P4HA3 is dispensable for prolyl 4-hydroxylation of type I collagen during mouse development
Source: J Biol Chem. 2026 Apr 16;302(6):111459. doi: 10.1016/j.jbc.2026.111459 (PMC13202288; doi:10.1016/j.jbc.2026.111459)
Supplement: Supporting information [file mmc1.pdf]

Supporting information

P4HA3 is dispensable for prolyl 4-hydroxylation of type I collagen during mouse development

Emma Karjalainen[1], Pekka Rappu[2], Valerio Izzi[1,3], Ilkka Miinalainen[4], Jyrki Heino[2], Johanna Myllyharju[1] , Antti M. Salo[1,*]

[1]Faculty of Biochemistry and Molecular Medicine, University of Oulu, Finland

[2]Department of Life Technologies, University of Turku, Finland

[3]Infotech Institute, University of Oulu, Finland

[4]Biocenter Oulu, University of Oulu, Finland

*Corresponding author, antti.salo@oulu.fi

List of materials

Table S1 Absolute amounts of total *P4ha* mRNA in postnatal tissues as copies/ng of input RNA.

Table S2 Sequencing results.

Table S3 Prolyl hydroxylation results by mass spectrometry

Table S4 Mass spectrometry results (as a separate excel spreadsheet)

Table S5 Sense- and antisense oligos of CRISPR/Cas9.

Table S6 PCR primers for CRISPR knockout screening.

Table S7 Primers used in qPCR analysis.

Fig. S1. Analysis of the relative expression of the P4ha isoforms in postnatal mouse tissues.

Fig. S2. *P4ha3* in situ hybridization by major systems and structures.

Fig. S3. Dynamics of *P4ha3* expression during embryonic development.

Fig. S4. *P4ha3* expression in skeletal, muscular and cartilage progenitors during embryonic development

Fig. S5. Sanger sequencing chromatograms of A) *P4ha3*$^{-/-}$ MEFs, B) *P4ha1*$^{-/-}$;*P4ha2*$^{-/-}$ MEFs, and C) *P4ha3*$^{-/-}$ MC3T3 cells.

Fig. S6. Uncropped Western blot image of MC3T3s.

Fig. S7. Uncropped Western blot membranes of MEFs.

Fig. S8. Uncropped SDS-PAGE gels of collagen extractions from MEFs and MC3T3s.

Fig. S9. Uncropped TEM images of MEFs and MC3T3

Table S1. Absolute amounts of total *P4ha* mRNA in postnatal tissues as copies/ng of input RNA. Data are presented as the mean value of combined (Bold) and individual *P4ha1, P4ha2* and *P4ha3* amounts, n=3–5 biological replicates per tissue. Green >500, yellow 100–500 and red <100 copies/ng of input RNA.

Total P4ha expression, copies/ng of input RNA

	**P0**	**P2**	**P4**	**1 wk**	**2 wk**	**6 wk**
**Tibia**[1]	**303,5** (169,7/76,7/57,1)	**799,8** (490,7/185,0/124,1)	**1155,0** (689,0/287,2/178,8)	**280,6** (194,1/74,0/12,5)	**59,2** (42,7/12,3/3,1)	**58,8** (45,0/11,7/2,2)
**Growth plate**[1]	**517,3** (196,4/111,1/209,8)	**676,2** (285,5/153,3/237,4)	**749,8** (375,9/164,3/209,6)	**223,4** (119,3/55,9/48,3)	**75,8** (38,0/27,1/10,8)	**0,2** (0,1/0,1/0,0)[2]
**Calvaria**	**498,8** (247,5/140,1/111,2)	**2635,3** (1540/720,7/374,7)	**888,8** (644,9/180,2/63,7)	**786,9** (487,7/208,7/99,5)	**341,7** (207,6/106,1/27,9)	**89,5** (62,6/21,1/5,8)
**Femur**						**15,0** (11,6/2,5/0,9)
**Femur distal epiphysis**						**0,2** (0,1/0,1/0,02)
**Bone marrow**						**2,5** (2,3/0,1/0,01)
**Lung**	**641,5** (419,6/115,5/106,4)	**1054,3** (661,0/161,5/231,8)	**599,9** (413,9/65,3/120,7)	**569,5** (235,8/44,8/288,99)	**81,6** (55,4/14,5/11,6)	**519,3** (388,7/118,3/12,3)
**Eye**	**432,4** (295,2/69,8/67,5)	**1064,4** (884,7/120,5/59,2)	**969,0** (776,0/130,0/63,0)	**509,7** (406,0/71,3/32,3)	**900,1** (742,7/132,5/24,9)	**32,1** (24,0/7,8/0,3)[3]
**Kidney**	**318,0** (238,4/40,2/39,4)	**613,8** (492,7/87,0/34,1)	**452,5** (331,0/88,4/33,1)	**517,6** (429,3/78,5/9,7)	**397,8** (319,5/75,9/2,4)	**268,7** (222,7/45,8/0,2)
**Brain**	**194,1** (123,9/33,5/36,8)	**527,9** (428,0/47,7/52,2)	**545,2** (414,3/64,3/66,9)			**217,0** (181,5/23,4/12,1)
**Heart**	**289,1** (130,4/77,2/81,4)	**731,6** (407,3/182,5/141,7)	**474,3** (278,1/120,3/75,9)	**76,4** (41,5/20,1/14,8)	**121,5** (69,9/50,5/1,1)	**386,7** (288,4/97,2/1,1)
**Aorta**						**73,6** (53,3/20,2/0,1)
**Microvascular endothelial cell**						**1168,2** (1024,0/137,8/6,4)
**Calf muscle**	**144,8** (76,2/22,8/45,8)	**858,7** (498/189,7/171,0)	**774,3** (422,2/163,6/188,5)	**259,5** (165,7/60,6/33,1)	**169,9** (109,9/55,5/4,6)	**88,6** (76,4/12,1/0,1)
**Skin**	**78,1** (44,4/25,3/8,3)	**99,7** (60,6/34,7/4,5)	**9,6** (8,0/1,5/0,1)	**35,6** (13,6/21,4/0,6)	**27,6** (21,1/6,4/0,1)	**48,9** (32,4/14,9/1,6)
**Liver**		**40,7** (32,9/5,1/2,8)	**35,8** (29,5/3,9/2,4)	**14,9** (13,3/1,0/0,6)	**42,6** (37,5/4,2/0,9)	**27,5** (22,5/4,9/0,1)
**Spleen**		**93,4** (81,5/4,1/7,9)	**100,8** (87,7/6,3/6,7)	**8,1** (7,3/0,3/0,5)	**11,3** (9,5/1,3/0,5)	**8,2** (8,0/0,18/0,05)
**Intestine**		**58,6** (45/8,7/4,8)	**8,9** (7,1/1,0/0,9)	**1,1** (0,8/0,2/0,0)		
**Tail tendon**					**987,7** (940,7,0/15,4/31,6)	**969,9** (960,4 /5,8/3,7)
**FDL tendon**						**65,1** (45,1/19,9/0,1)
**Achilles tendon**						**8,6** (5,2/3,0/0,4)
**Xiphoid**						**65,1** (45,4/17,0/2,6)

[1]Tolonen et al. 2022, [2]tibia proximal epiphysis, [3]cornea

Table S2. Sequencing results

				Genomic variant		Protein level consequence		
**cell line**	**genotype before editing**	**gDNA target**	**clone**	**allele 1**	**allele 2**	**allele 1**	**allele 2**	**knockout**
MEF	WT	P4ha3-exon2	4	290insC	290insC	*W98fs*	*W98fs*	P4ha3$^{-/-}$
MEF	WT	P4ha3-exon2	7	290insC	290insC	*W98fs*	*W98fs*	P4ha3$^{-/-}$
MEF	WT	P4ha3-exon2	10	290insC	290insC	*W98fs*	*W98fs*	P4ha3$^{-/-}$
MEF	P4ha1$^{-/-}$	P4ha2-exon3	1	239delG	230_233delACCC	*G80fs*	*D77fs*	P4ha1$^{-/-}$;P4ha2$^{-/-}$
MEF	P4ha1$^{-/-}$	P4ha2-exon3	6	234delC	233_234delCC	*P78fs*	*P78fs*	P4ha1$^{-/-}$;P4ha2$^{-/-}$
MEF	P4ha1$^{-/-}$	P4ha2-exon3	10	234delC	228_240del	*P78fs*	*A76fs*	P4ha1$^{-/-}$;P4ha2$^{-/-}$
MC3T3	WT	P4ha3-exon2	4	292insT		*W98fs*		*P4ha3*$^{-/-}$
MC3T3	WT	P4ha3-exon2	5	292insT	291_295delCTGGA	*W98fs*	*D97fs*	*P4ha3*$^{-/-}$
MC3T3	WT	P4ha3-exon2	6	291delC		*D97fs*		*P4ha3*$^{-/-}$
MC3T3	WT	P4ha3-exon2	8	288delC_290A>G		*S96fs*		*P4ha3*$^{-/-}$
MC3T3	WT	P4ha3-exon2	9	296insG	293insC_294delGG	*R99fs*	*W98fs*	*P4ha3*$^{-/-}$

Table S3. Prolyl hydroxylation results by mass spectrometry

Sample	OG or PG	OG not PG	OG not PG / OG or PG
P4ha3-/-	1618	1302	0.80
P4ha3-/-	1590	1228	0.77
P4ha3-/-	1510	1144	0.76
WT	1404	1038	0.74
WT	1737	1344	0.77
WT	1329	968	0.73
WT	1321	1001	0.76
WT	1578	1213	0.77
P4ha1-/-	1364	590	0.43
P4ha1-/-	1351	635	0.47
P4ha1-/-	1338	586	0.44
P4ha1-/-	1281	640	0.50
P4ha1-/-	1297	573	0.44

Table S4 Mass spectrometry results

see separate excel spreadsheet

Table S5. Sense- and antisense oligos of CRISPR/Cas9

oligo name	Sequence (5´-3´)
P4ha2 guide ex-3-sense	CACCGAGATCAGCTGCCGACCCCGA
P4ha2 guide ex-3-antisense	AAACTCGGGGTCGGCAGCTGATCTC
P4ha3 guide ex-2-sense	CACCGAACGTCTGCAGTCCGACTGG
P4ha3 guide ex-2-antisense	AAACCCAGTCGGACTGCAGACGTTC

Table S6. PCR primers for CRISPR knockout screening.

oligo name	Sequence (5´-3´)
P4ha2 ex3 gDNA F	TGAGAGTTCCCAGCTCTTACC
P4ha2 ex3 gDNA R	CCAAATCTGGATCCTCGGCA
P4ha3 ex2 gDNA F	AGCCAGCTCCTGATTTTAGCA
P4ha3 ex2 gDNA R	GGAAGGCCATGATGTTTTGCA

F: Forward R: Reverse

Table S7. Primers used in qPCR analysis

Gene	Forward sequence (5´--> 3´)	Reverse sequence (5´--> 3´)
**Tbp**	GAATATAATCCCAAGCGATTTG	CACACCATTTTTCCAGAACTG
**Actb**	AGAGGGAAATCGTGCGTGAC	CAATAGTGATGACCTGGCCGT
**Gapdh**	TGTGTCCGTCGTGGATCTGA	TTGCTGTTGAAGTCGCAGGAG
**P4ha1**	ATCTCTGACGCGGAGATTGAG	CAGGGTCTTCGTAGCCAGACA
**P4ha2**	GTCCTGGTTCGGTGTCCTGAGC	CAGATCGGTCATGTGCCCAATGG
**P4ha3**	AGGCCCAACGTACCCCACCT	GTGTGTTGGCTGGGAGCCCA
**Plod1**	GATGTCTACTGGTTCCCCATCTTC	CCTGGATCCGGTTGTCCTTATTAT
**Plod2**	GCCAGTGGCAATTAATGGAA	CCTGTGTCCATGAGTTTGGA
**Plod3**	CCTTCACAACAGCGAGGTGTA	CTCTGGCCCCACCAGCTTTA
**Lox**	QuantiTect Mm_Lox_1_SG(QT00098028)	
**Col1a1**	TGTGTGCGATGACGTGCAAT	GGGTCCCTCGACTCCTACA
**Col1a2**	AAGGGTGCTACTGGACTCCC	TTGTTACCGGATTCTCCTTTGG
**Col3a1**	CTGTAACATGGAAACTGGGGAAA	CCATAGCTGAACTGAAAACACC
**Col4a1**	TCCCGGACTTCCTGGTATT	CCTTTGTACCGTTGCATCCT
**Col4a2**	AGAAAGGAGCAAGGGGTCAG	CTTTGCGCCCTGTAGTC
**Col4a3**	GGGAGTGGCAAGAAGTATGG	CGAGATCCCTTCTCAGGAAA
**Col4a4**	GGGAGTGGCAAGAAGTATGG	CGAGATCCCTTCTCAGGAAA
**Col4a5**	ACGTGGATTTCCAGGCAGT	CTGGTTCCCCCTTCATACCT
**Col9a1**	ACCGACCAGCACATCAA	AGGGGGACCCTTAATGC
**Col12a1**	AAGTTGACCCACCTTCCGAC	GGTCCACTGTTATTCTGTAACCC
**Col14a1**	CCCCAGAATAGAGTGGCACTT	CAAAGCAAGACCTGTTAGGGTAT
**Col20a1**	GTCCGACACCTGACTTTCTCA	GCTGAAGTAAGGTTCCCAGGA
**Fn1**	ATGTGGACCCCTCCTGATAGT	GCCCAGTGATTTCAGCAAAGG

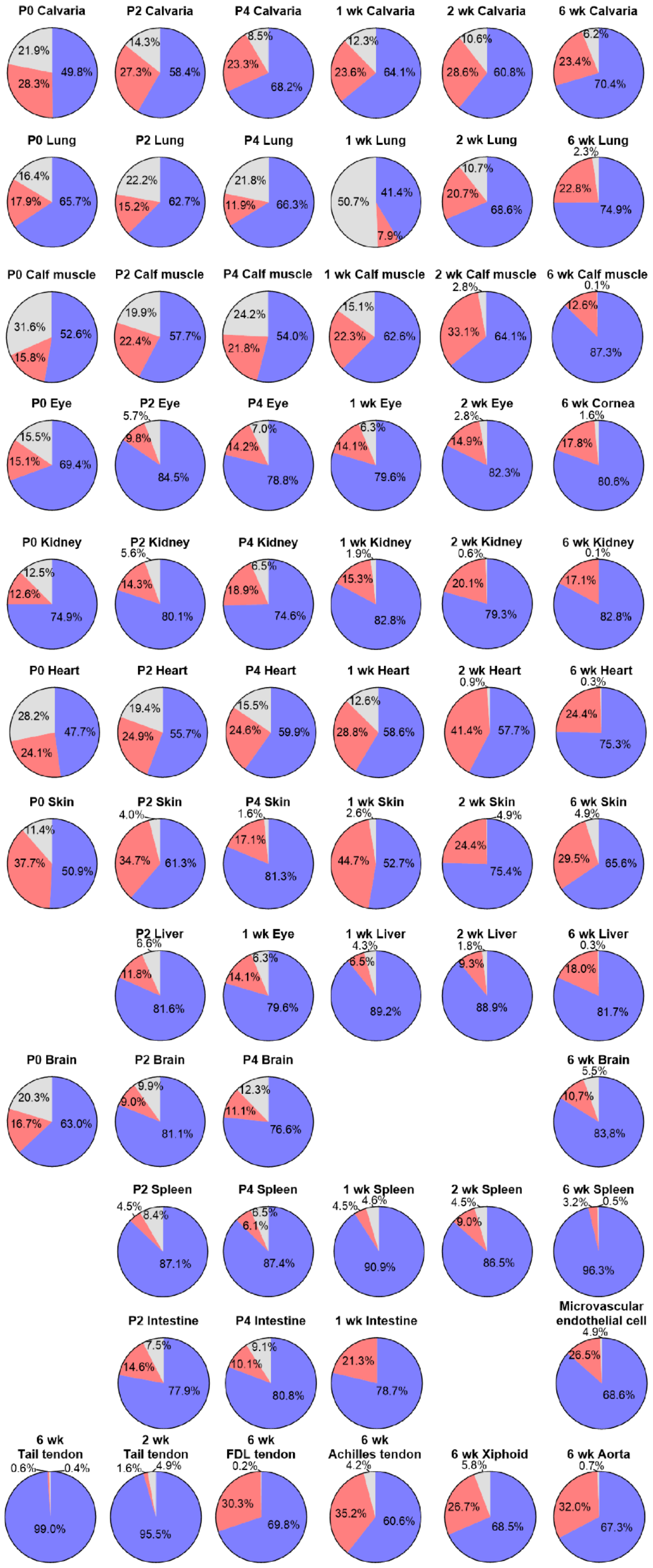

Fig. S1. Analysis of the relative expression of the P4ha isoforms in postnatal mouse tissues. The data used in the analysis are from Fig. 2.

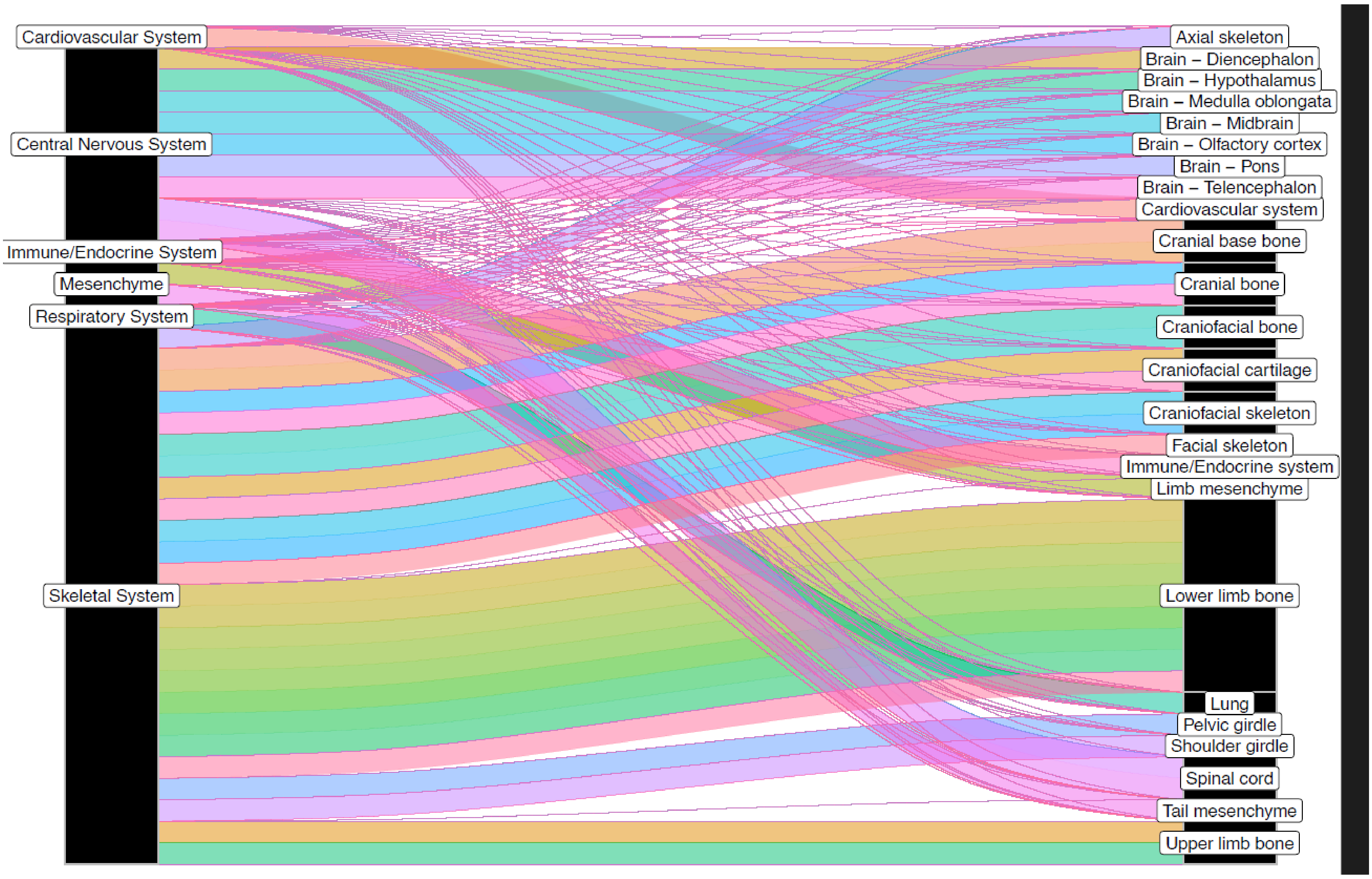

Fig. S2. *P4ha3* in situ hybridization by major systems and structures. Relative quantification of *P4ha3* in situ hybridization (ISH) signal by EMAGE systems and anatomical regions' annotations.

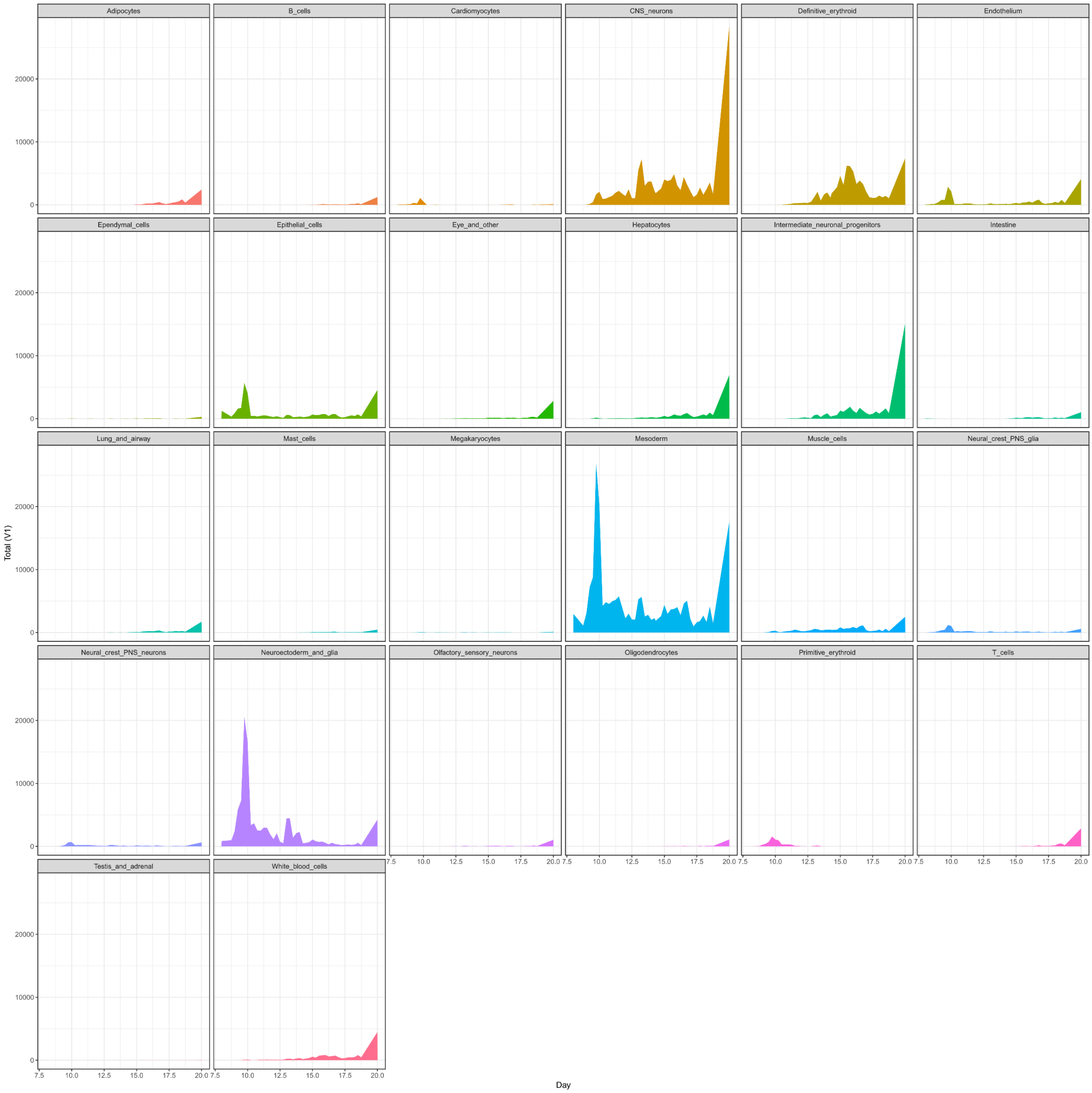

Fig. S3. Dynamics of *P4ha3* expression during embryonic development. Plots show the expression of *P4ha3* (total counts per cell type) across development day, from gastrula to birth.

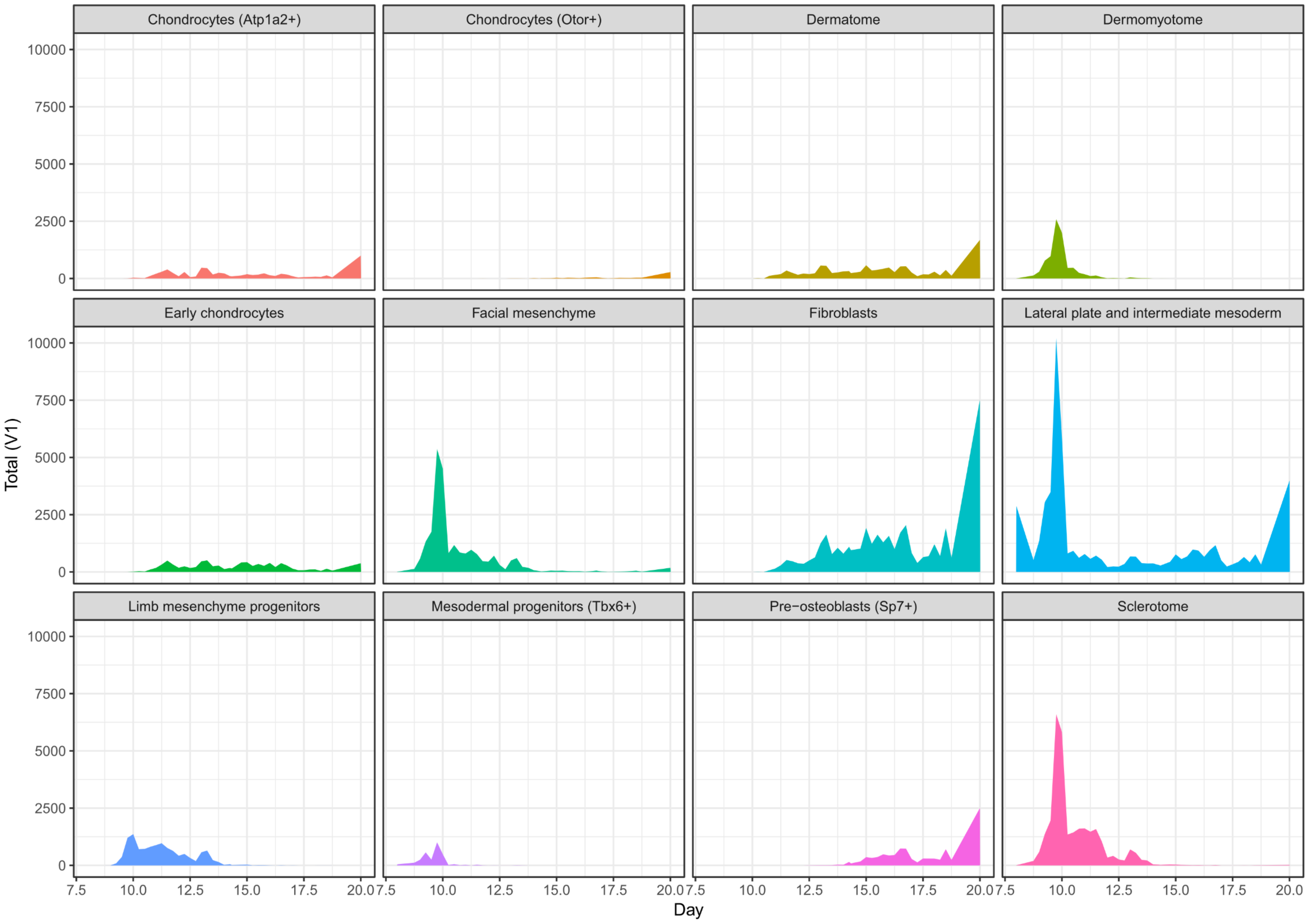

Fig. S4. *P4ha3* expression in skeletal, muscular and cartilage progenitors during embryonic development. Plots show the expression of *P4ha3* (total counts per cell type) across development day, from gastrula to birth.

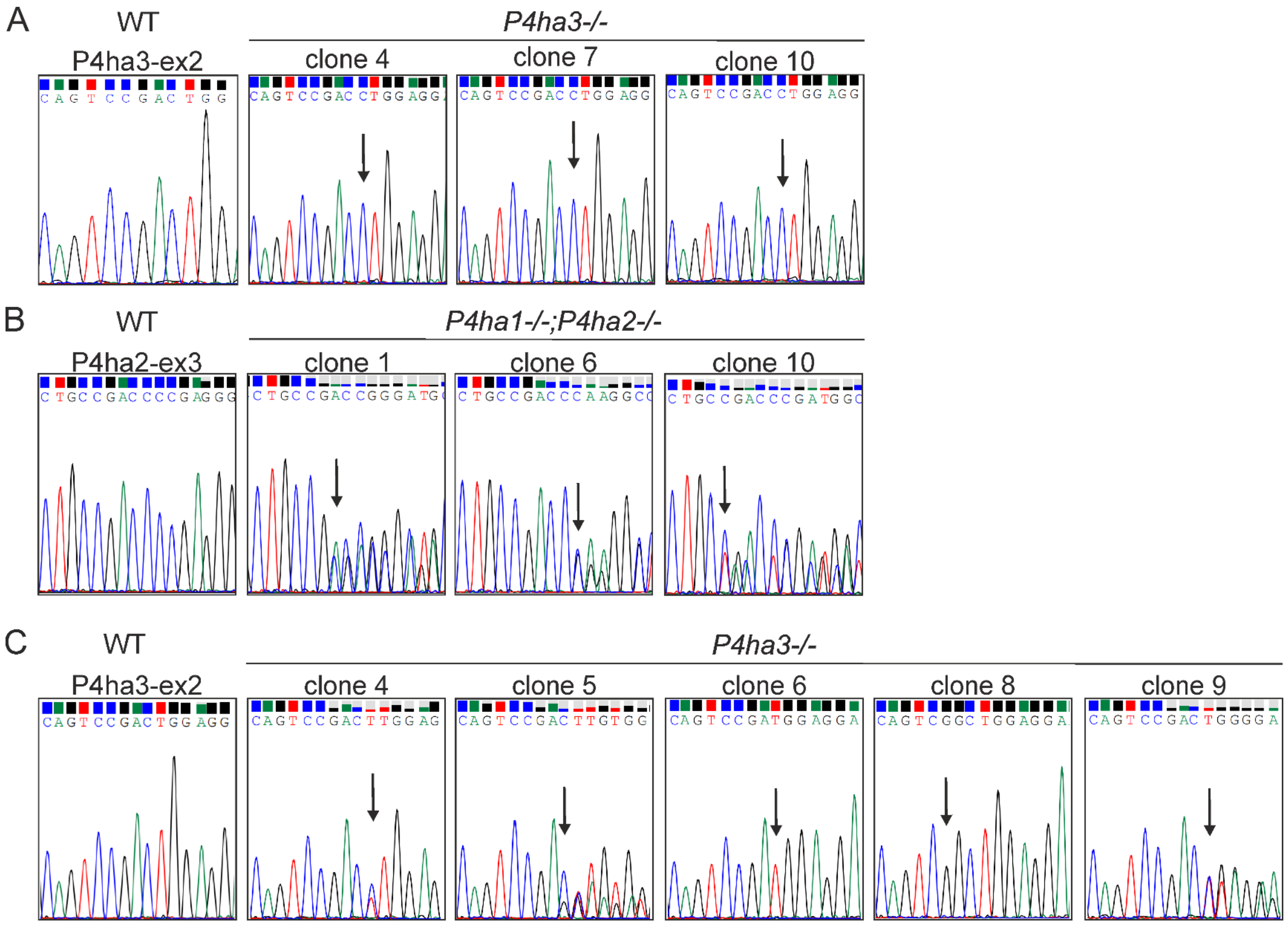

Fig. S5. Sanger sequencing chromatograms of A) *P4ha3*$^{-/-}$ MEFs, B) *P4ha1*$^{-/-}$;*P4ha2*$^{-/-}$ MEFs, and C) *P4ha3*$^{-/-}$ MC3T3 cells. Arrows indicate the targeted site, where the gene edit has occurred. All clones have a frameshift mutation that lead to premature stop codon.

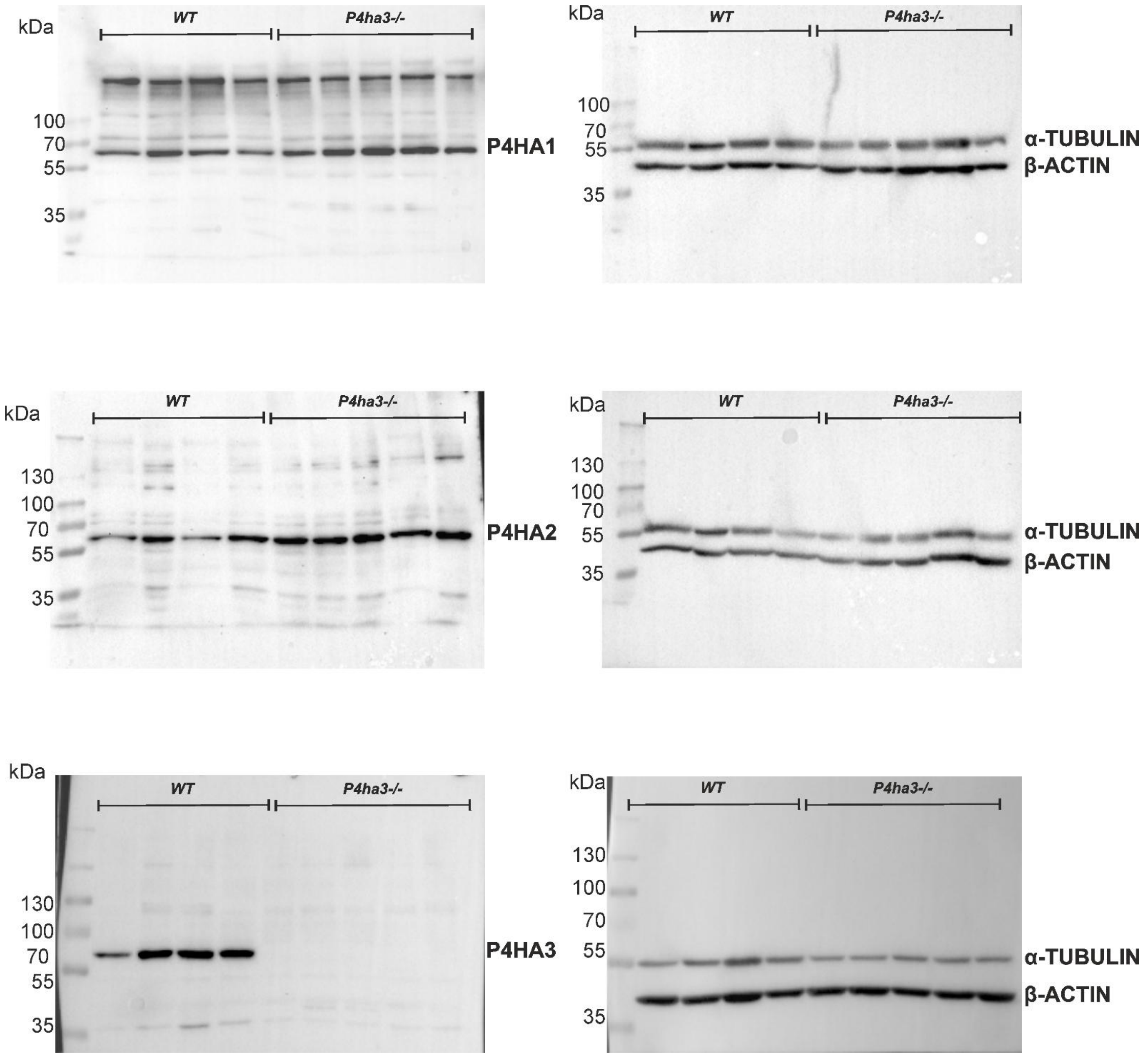

Fig. S6. Uncropped western blot images of MC3T3s. Whole blot image that was used in Figure 3C.

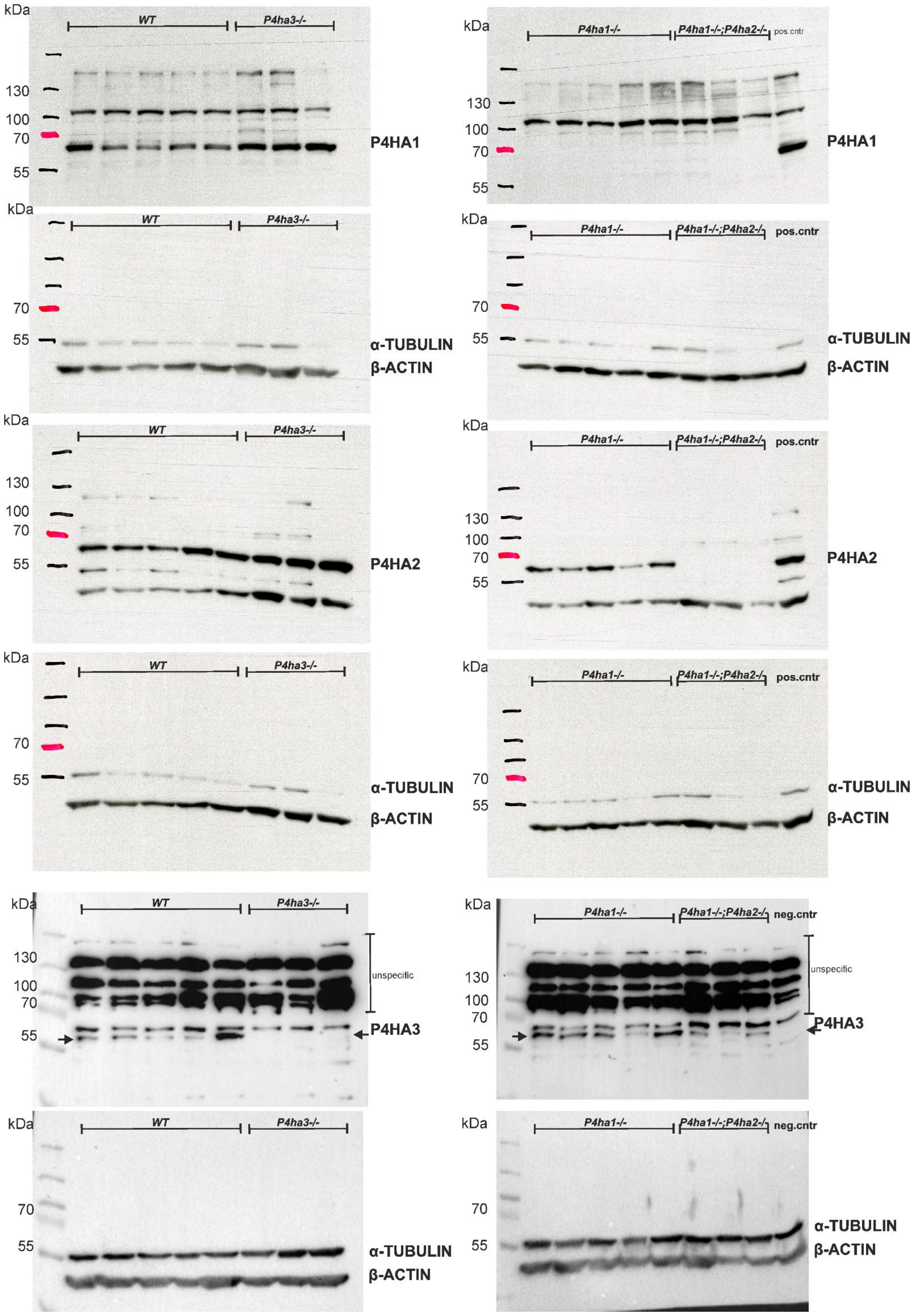

Fig. S7. Uncropped Western blot membranes of MEFs. Whole blot image that was used in Figure 3B.

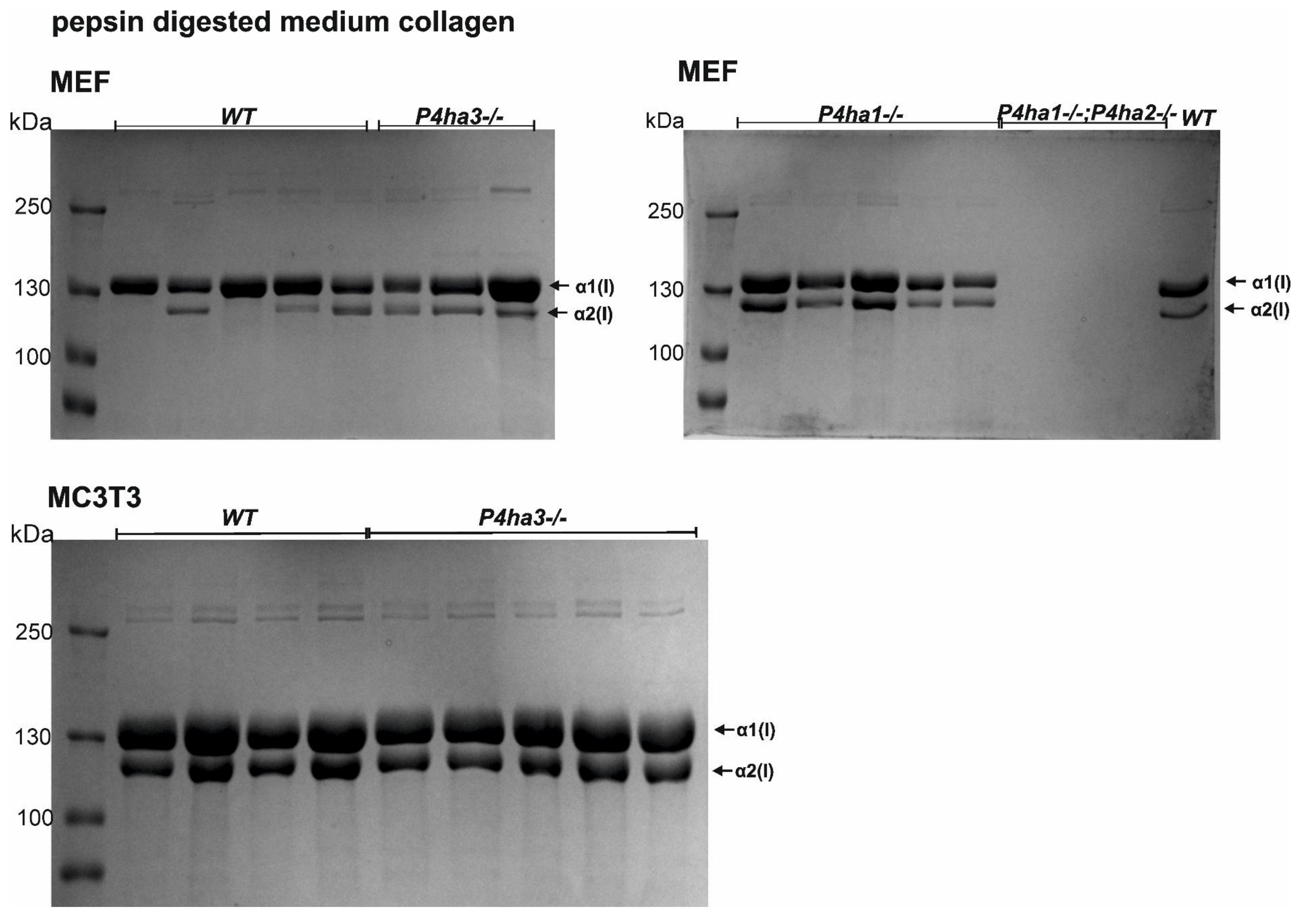

Fig. S8. Uncropped SDS-PAGE gels of collagen extractions from MEFs and MC3T3s. Whole SDS-PAGE image that was used in Figure 6A and 6B.

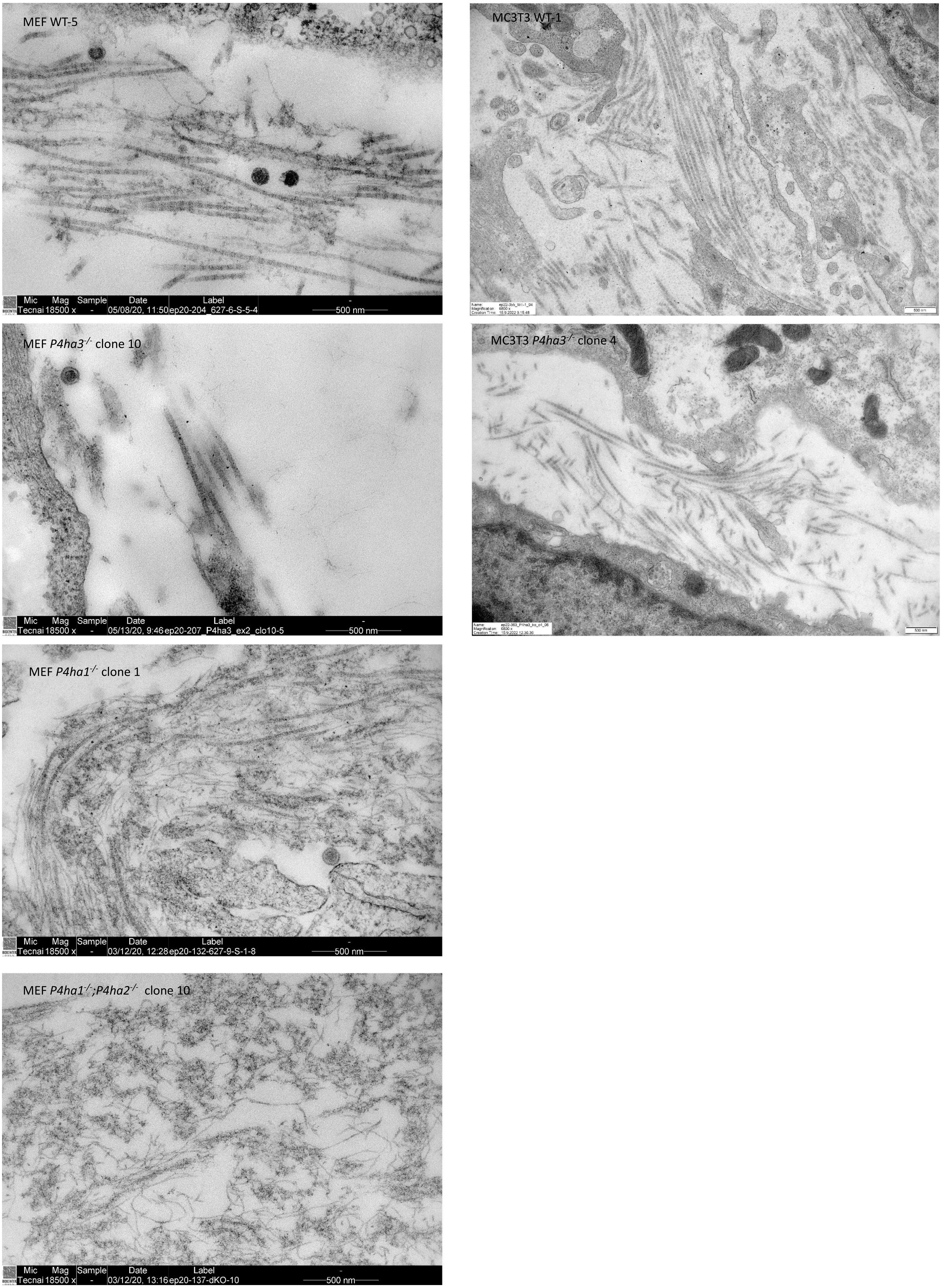

Fig. S9. Uncropped TEM images of MEFs and MC3T3s.
